# The neural stem cell fate determinant TRIM32 regulates complex behavioral traits

**DOI:** 10.3389/fncel.2015.00075

**Published:** 2015-03-18

**Authors:** Anna-Lena Hillje, Elisabeth Beckmann, Maria A. S. Pavlou, Christian Jaeger, Maria P. Pacheco, Thomas Sauter, Jens C. Schwamborn, Lars Lewejohann

**Affiliations:** ^1^ZMBE, Institute of Cell Biology, Stem Cell Biology and Regeneration Group, Westfälische Wilhelms-Universität MünsterMünster, Germany; ^2^Luxembourg Centre for Systems Biomedicine, University of LuxembourgLuxembourg, Luxembourg; ^3^Department of Behavioural Biology, Westfälische Wilhelms-Universität MünsterMünster, Germany; ^4^Life Sciences Research Unit, University of LuxembourgLuxembourg, Luxembourg

**Keywords:** adult neurogenesis, cell fate determinant, neural stem cells, olfactory behavior, brain metabolism

## Abstract

In mammals, new neurons are generated throughout the entire lifespan in two restricted areas of the brain, the dentate gyrus (DG) of the hippocampus and the subventricular zone (SVZ)—olfactory bulb (OB) system. In both regions newborn neurons display unique properties that clearly distinguish them from mature neurons. Enhanced excitability and increased synaptic plasticity enables them to add specific properties to information processing by modulating the existing local circuitry of already established mature neurons. Hippocampal neurogenesis has been suggested to play a role in spatial-navigation learning, spatial memory, and spatial pattern separation. Cumulative evidences implicate that adult-born OB neurons contribute to learning processes and odor memory. We recently demonstrated that the cell fate determinant TRIM32 is upregulated in differentiating neuroblasts of the SVZ-OB system in the adult mouse brain. The absence of TRIM32 leads to increased progenitor cell proliferation and less cell death. Both effects accumulate in an overproduction of adult-generated OB neurons. Here, we present novel data from behavioral studies showing that such an enhancement of OB neurogenesis not necessarily leads to increased olfactory performance but in contrast even results in impaired olfactory capabilities. In addition, we show at the cellular level that TRIM32 protein levels increase during differentiation of neural stem cells (NSCs). At the molecular level, several metabolic intermediates that are connected to glycolysis, glycine, or cysteine metabolism are deregulated in TRIM32 knockout mice brain tissue. These metabolomics pathways are directly or indirectly linked to anxiety or depression like behavior. In summary, our study provides comprehensive data on how the impairment of neurogenesis caused by the loss of the cell fate determinant TRIM32 causes a decrease of olfactory performance as well as a deregulation of metabolomic pathways that are linked to mood disorders.

## Introduction

Adult neurogenesis has been reported in the mammalian brain in two regions, the subventricular zone (SVZ) located in the wall of the lateral ventricles and the dentate gyrus (DG) of the hippocampus (Gage, 2000).

In the SVZ, neural stem cells (NSCs), also called type B cells, are astrocytes that are able to self-renew and at the same time give rise to transit amplifying cells (type C cells) (Doetsch et al., 1999). These transient amplifying cells differentiate into neuroblasts (type A cells) that migrate along the rostral migratory stream (RMS) to the olfactory bulb (OB) via chain migration (Doetsch et al., 1997). In the OB they finally become mature neurons which are integrated into the neuronal network (Belluzzi et al., 2003; Carleton et al., 2003). Adult newborn neurons turn into OB interneurons; i.e., granule cells and periglomerular cells (Petreanu and Alvarez-Buylla, 2002). Granule cells of the OB shape the information passed from the projecting cells of the bulb (mitral and tufted cells) on to higher brain areas (Nissant and Pallotto, 2011). Thereby, they are able to spatiotemporally shape the mitral cell signal in a process called lateral inhibition. This process is supposed to facilitate odor encoding and discrimination (Yokoi et al., 1995; Urban, 2002; Tan et al., 2010; Ernst et al., 2014).

In the hippocampus, type I NSCs reside the inner layer of the DG, the subgranular zone (reviewed in Yao et al., 2012). They generate self-amplifying type II intermediate progenitor cells, which migrate to the outer layers of the DG (Kuhn et al., 1996). Type II cells eventually give rise to type III neuroblasts that differentiate into glutamatergic dentate granule cells (DGCs) (Kuhn et al., 1996). Adult born DGCs integrate into the existing network and form synaptic connections with the entorhinal cortex and the CA3 subfield (van Praag et al., 2002; Toni et al., 2007; Yao et al., 2012). This facilitates adult born neurons to contribute to pattern separation, a process that allows the distinct encoding of very similar stimuli (reviewed in Vivar and van Praag, 2013). Additionally, hippocampal adult neurogenesis has been shown to be involved in the regulation of cognition and mood (Zhao et al., 2008) as well as learning and memory (reviewed in Stuchlik, 2014).

Adult born neurons display unique properties that clearly distinguish them from mature neurons (reviewed in Ming and Song, 2011). After forming synaptic connections, newborn neurons in both, hippocampus and OB, show enhanced excitability as well as increased synaptic plasticity at certain stages of neuronal maturation (Nissant et al., 2009; Ming and Song, 2011). By this, they are able to modulate the existing local circuitry of established mature neurons and add specific properties to information processing (Bardy et al., 2010). Many studies aimed at investigating the behavioral functions of adult neurogenesis. In these studies a variety of different methods were used to alter cell turnover rates in the SVZ (reviewed in Nissant and Pallotto, 2011). This methodological variation might explain that the results vary considerably with regards to effects on learning and memory. For example, differences in spontaneous odor discrimination, associative learning tasks as well as in short-term and in long-term memory have been reported (Lazarini and Lledo, 2011; Breton-Provencher and Saghatelyan, 2012).

The TRIM-NHL protein family has an evolutionary conserved function in neuronal cell fate specification in *C. elegans*, Drosophila, and mammals (Betschinger and Knoblich, 2004; Bello et al., 2006; Lee et al., 2006; Hammell et al., 2009; Schwamborn et al., 2009; Hillje et al., 2011). During embryonic development of the neocortex in mice, the TRIM-NHL protein TRIM32 regulates cell fate decisions of newly born daughter cells (Schwamborn et al., 2009). In the adult brain, TRIM32 is upregulated during differentiation of SVZ generated neuroblasts and is necessary for the correct induction of neuronal differentiation of these cells (Hillje et al., 2013). Loss of TRIM32 results in an overproduction of adult generated OB neurons, which is the combined result of increased progenitor proliferation and decreased apoptosis.

On the molecular level, TRIM32 induces neuronal differentiation and suppresses self-renewal by ubiquitination of the transcription factor c-Myc and the activation of certain microRNAs (Schwamborn et al., 2009; Nicklas et al., 2012). TRIM32 has been linked to several human diseases including limb–girdle muscular dystrophy type 2H (Frosk et al., 2002, 2005; Kudryashova et al., 2005, 2012), Bardet–Biedl syndrome (Chiang et al., 2006), cancer (Horn et al., 2004; Kano et al., 2008), autism spectrum disorder (Lionel et al., 2011, 2014), depression (Ruan et al., 2014), Alzheimer's disease (Yokota et al., 2006), obsessive compulsive disorder (Lionel et al., 2014), anxiety (Lionel et al., 2014), and attention deficit hyperactivity disorder (Lionel et al., 2011, 2014).

In the here presented study we show that the absence of the cell fate determinant TRIM32 alters the performance of mice in an olfactory habituation task without influencing the long-term olfactory memory. In order to control for inadvertent influence of other behavioral traits, we included a variety of tests investigating DG-related behavior in our study. Finally, we have hints that an impairment of adult neurogenesis caused by loss of TRIM32 results in the deregulation of metabolomic pathways that have been linked to depression and anxiety related behavior. Altogether, our study provides comprehensive data on how the impairment of neurogenesis caused by the loss of the cell fate determinant TRIM32 leads to decreased olfactory performance as well as changes in metabolomic profiles.

## Materials and methods

### Animals and housing conditions

A total of 14 male TRIM32 knockout mice and 21 male wildtype litter mates were used. All mice were born and tested in the Department of Behavioural Biology, University of Muenster. Their parents were derived from a locally bred colony at the Center for Molecular Biology of Inflammation that was founded from cryo-preserved spermatozoa derived from the Mutant Mouse Regional Research Centers, USA. Gene knockout of TRIM32 has been described earlier (Kudryashova et al., 2009). In detail, T32KO mice were generated using the BGA355 mouse embryonic stem cell line [BayGenomics (former web-site https://www.mmrrc.org/catalog/sds.php?mmrrc_id=11810; now available at International Gene Trap Consortium, http://www.genetrap.org/cgi-bin/annotation.py?cellline=BGA355)] carrying gene trap insertion in TRIM32, within exon 2. The position of the integration site was confirmed after nucleotide 278 starting from the ATG codon in exon 2 of the *trim32* gene. Original founder mice were 129 SvEvBrd × C57 BL/6 chimeras, which were backcrossed to C57 BL/6J wt mice to obtain germ line transmission. Heterozygotes from this cross were interbred to produce ko and wt homozygotes (Kudryashova et al., 2009). All analyses were performed on interbred mice on a mixed 129 SvEvBrd × C57 BL/6J background. To ascertain a congenic background more than seven backcrosses were done.

All animals were housed in a temperature controlled room at 22°C and a relative humidity of 45% ± 10%. A 12-h dark-light circle with lights on at 8.00 a.m. was installed. Offspring were weaned at postnatal day 22 and experimental mice were kept in standard cages (37 × 21 × 15 cm) in groups of 3–5 animals, preferably in groups of littermates. Tissue for genotyping was sampled by ear cuts and genotype specific DNA fragments were identified after PCR amplification and agarose gel electrophoresis. Ear-cuts also allowed individual discrimination of mice from the same cage. However, behavioral experiments were carried out with the experimenter being unaware of the genotypes of the subjects. Food (Altromin 1324, Altromin GmbH, Lage, Germany) and water were available *ad libitum*. A thin layer of wood shavings and paper towels served as bedding and nesting material that was changed weekly while transferring the mice to clean cages.

All procedures and protocols met the guidelines for animal care and animal experiments in accordance with national and European (86/609/EEC) legislation.

### Health check

To determine whether all mice included in the testing were healthy a health check was performed. In order to prevent interference of the behavioral tasks with testing experience gained during the health check, the test was conducted at postnatal day 101 and thus would have allowed retrospectively excluding animals with visible or detectable bodily defects. However, none of the tested animals had to be excluded. Health parameters were tested according to Lewejohann et al. (2004) and included general appearance (e.g., fur, ears, eyes, vibrissae, extremities, and tail), sensory abilities (e.g., vision, startle response, tactile reaction), reflex functions (e.g., eyelid reflex, grasp reflex), and locomotor/coordinative abilities (climbing, balancing).

It was previously observed that male TRIM32 knockout mice weighted more than wildtype mice after reaching an age of 8 weeks (Kudryashova et al., 2009). We therefore measured weight development of all mice beginning at an age of 3 weeks until the age of 15 weeks (Supplementary Figure 1). However, under the here applied experimental conditions we were unable to detect a significant increase in weight in aging TRIM32 knockout mice.

### Elevated Plus Maze (EPM)

The EPM was conducted with 14 TRIM32 knockout and 20 wildtype male mice at an age of 65 (±2) days of age. The test measures anxiety related behavior by exploiting the tendency of mice to avoid exposed areas in favor of shielded areas. The apparatus consists of four 30 × 5 cm arms emerging from a central platform of 5 × 5 cm. Two opposing arms are open while the two orthogonal arms are enclosed by walls 20 cm of height. The apparatus was elevated ca. 50 cm above the ground. The runway of the open arms was surrounded by a low balustrade (0.5 cm), effectively preventing the mice from jumping or falling off. On the ceiling above the apparatus, at a height of 175 cm, a camera (Logitech Pro 9000, Freemont, USA) was installed, as well as a light bulb emitting ca. 100 lux. Videos of the mice performing the test were recorded and subsequently analyzed using an in-house programmed animal tracking software (Lewejohann et al., 2004). Before testing started, the runways and walls of the maze were cleaned with 70% ethanol to remove possible olfactory cues from preceding tested mice. In order to control for a similar level of alertness, the mice were placed in an empty cage for 1 min prior to testing. Each mouse was then placed on the central platform facing one of the closed arms. After 5 min of freely exploring the apparatus, the recording was stopped and the mouse was transported back into its home cage.

The parameters which were analyzed included the total path length in meters, time spent in closed and open alleys in seconds, as well as the number of entries into the open and closed arms.

### Open field test (OF)

The OF was conducted with 14 TRIM32 knockout and 21 wildtype male mice at an age of 67 (±2) days. The test evaluates locomotor activity and exploratory behavior in an open box measuring 80 by 80 cm with surrounding walls of 40 cm height. Comparable to the EPM test anxiety related behavior (avoidance of unprotected areas) can be observed by measuring the time spent in the center of the box in relation to the time spent in the more protected areas close to the walls of the box. The box was lit at ca. 100 lux and a camera was placed centrally above the apparatus. The apparatus was cleaned with 70% ethanol before testing and the mice were placed in an empty cage 1 min prior to being placed in the center of the open field. Videos of the mice performing the test were recorded for 5 min and subsequently analyzed (see EPM). The parameters analyzed included path length, time in center, time close to the wall, number of stops, and velocity. Stops were recognized by the tracking software when the velocity was 0 for at least 1 s.

### Barnes Maze (BM)

The Barnes Maze was first developed by Barnes (1979) to compare spatial learning abilities of young vs. senescent rats. The maze itself consists of a circular platform of 1 m in diameter with 12 holes at the circumference drilled in an equal distance from each other. One of the holes is chosen to be the target hole for the tested individual and connected to the animal's home cage. The platform was brightly lit in order to create a mildly aversive environment that was escapable by learning the position of the correct hole during the course of repeated trials. Around the platform, visual cues were placed in order to facilitate spatial orientation.

During the training phase, one of the holes was connected to the animal's home cage via a wire-mesh tunnel, while all other holes were closed by a short tube made of wire mesh. Choosing the same ventilatable wire mesh for the rewarded as well as for the unrewarded holes guaranteed that it was not possible for the mouse to discriminate between open and closed holes from above. The maze was raised 125 cm above the floor and illuminated by a 100 W electric bulb located 110 cm above the center of the maze.

Two training trials with an inter-trial interval (ITI) of 1 h were conducted on four consecutive days with 14 TRIM32 knockout and 21 wildtype male mice starting at an age of 72 (±2) days. On the fifth day a probe trial testing spatial memory was conducted with all holes being closed for 5 min. During this trial it was measured whether or not the animal spent significantly more time in the sixth of the BM were formally the escape hole was located. After the probe trial an additional training trial with the correct hole being connected to the home cage again was done in order to reinstall the memory for the correct position of the hole. One week later a single re-test was conducted in order to test for intermediate to long-term spatial memory. Before each trial the BM was cleaned with 70% ethanol and all mice were placed for 1 min in a starting cylinder placed in the middle of the platform. All trials were video recorded and analyzed using an animal tracking software (see EPM). For each trial, the time the mice needed to find the target holes, number of errors, as well as the path length traveled was measured.

### Olfactory habituation test (OH)

To test olfactory discriminative abilities and short-term memory for different odors an olfactory habituation/dishabituation task was chosen. The odorants used were (A) isoamyl-acetate (SAFC, Hamburg, Germany), (B) 4-methylcyclohexanol (Sigma Aldrich, Steinheim, Germany), and (C) 3-octanol (Sigma Aldrich, Steinheim, Germany). All odorants were diluted 1:100 in paraffin oil (Sigma Aldrich, Steinheim, Germany). These odorants are described as being perceived dissimilar (Mandairon et al., 2006) and in a preliminary experiment, C57BL/6J wild-type mice did not show a preference for any of these odors when being exposed to them in an open field apparatus (data not shown).

The OH was conducted at an age of 80 (±2) days with 14 TRIM32 knockout and 18 wildtype male mice accordingly to the protocol by Yang and Crawley (2009) with slight modifications. For each trial the mouse was placed into a clean cage (Macrolon type I, 19 × 10 × 13 cm) filled with fresh bedding. In the center of the cage lid, a wooden cotton swab (15 cm, PARAM GmbH, Hamburg, Germany) was attached so that its tip reached into the cage for ca. 7 cm. In order to acclimate to the new environment, the mouse was left undisturbed in the cage for 25–30 min. The cage was then transferred to a chamber that was directly connected to the air ventilation system of the animal housing facility in order to minimize surrounding odors and also to eliminate any scents previously applied as fast as possible.

Each tested mouse was firstly exposed to distilled water and subsequently to three different odors (the order of odors was randomized for each mouse). Each substance was applied 3 times in a row with an inter trial interval of 30 s. The experimenter observed the behavior for 90 s and measured the time the mouse spent sniffing on the cotton tip (snout closer than 2 cm), the latency of the first sniff, and the number of sniffs. The test was repeated after 10 days in order to measure long-term olfactory memory.

### Injection of bromodeoxyuridine (BrdU)

Animals were injected with 50 mg/kg BrdU on 3 consecutive days and sacrificed after the indicated time. Sections were incubated with 2 M HCl in PBST (PBS+0.3% Triton) for 25 min at 37°C for denaturation of the DNA, neutralized with 0.1 M sodium tetraborate (pH 8.5) for 7 min at room temperature and stained with an anti-BrdU antibody (AbDSerotec). On day 107–109 at 12 a.m., the mice received a BrdU injection and at day 123, the animals were sacrificed. The brains were dissected and analyzed (Hillje et al., 2013). For quantification of BrdU+ cells in the DG, two sections each of 5 wt and 5 TRIM32 ko brains were analyzed, for quantification of BrdU+ cells in the OB, two sections of 4 wt and 4 TRIM32 ko mice were used. In each case, the mean of BrdU+ cells in TRIM32 ko tissue was normalized to the mean of BrdU+ cells in sections of wt brains.

### Immunohistochemistry of free-floating sections

**Figure 1:**
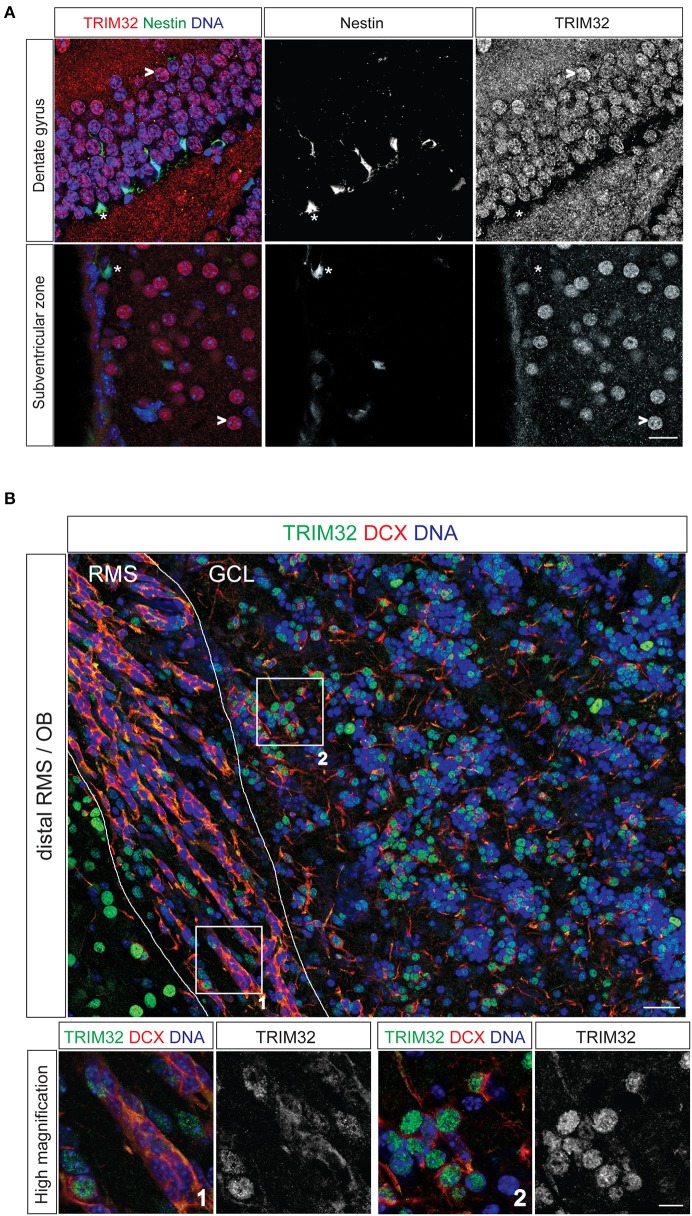
**TRIM32 is upregulated upon neuronal differentiation of subventricular zone (SVZ) and dentate gyrus (DG) stem cells**. Free floating sections from adult mouse brain stained with the indicated antibodies. **(A)** Free floating sections from adult Nestin-GFP mice stained with the indicated antibodies. (^*^) highlights neural stem cells in the DG and SVZ, (>) marks mature neurons. Scale bar = 20 μm. **(B)** Free floating sections from wt mice stained with the indicated antibodies. Images in the lower panel represent high magnification of the indicated areas labeled in upper image. Scale bar = 30 μm for upper image, 10 μm for lower panel. RMS, rostral migratory stream; GCL, granular cell layer; OB, olfactory bulb.

Mice were deeply anesthetized by intraperitoneal injection of 0.017 ml of 2.5% Avertin (100% stock solution: 10 g 2, 2, 2-Tribromoethanolin 10 ml tert-Amylalcohol) per gram of body weight and sacrificed by perfusion. Brains were fixed overnight at 4°C in 4% paraformaldehyde in phosphate buffer saline (PBS). Later, sections of 40 μm were prepared using a vibratome (Leica, Wetzlar, Germany) and blocked for at least 1 h in TBS (0.1 M Tris,150 mM NaCl, pH 7.4) containing 0.5% Triton X 100, 0.1% Na-Azide, 0.1% Na-Citrate, and 5% normal goat serum. Immunostainings were performed by incubation of the sections with primary antibodies diluted in the blocking solution for 48 h at 4°C on a shaker, followed by incubation with the secondary antibody diluted in the blocking solution for 2 h at room temperature. Finally, sections were mounted in AquaMount (DAKO, Glostrup, Denmark). The following primary antibodies were used for immunohistochemistry: anti-Neuronal Nuclei (NeuN) (mouse, Millipore), anti-Doublecortin X (guinea pig, Millipore), anti-TRIM32-1137 (Figure 1A, rabbit, Gramsch Laboratories, Schwabhausen, Germany), anti-TRIM32-GS (Figure 1B, rabbit, Gramsch Laboratories, Schwabhausen, Germany). As secondary antibodies Alexa goat anti-rabbit-568, Alexa goat anti-rabbit 568, Alexa goat anti-mouse 488, Alexa goat anti-mouse 568, and Alexa goat anti-guinea pig 568 (all from Invitrogen) were used. Nuclei were stained using Hoechst 33342 (Invitrogen). Images were collected by confocal microscopy using ZEN software (Zeiss, Jena, Germany); image analysis was performed with the ZEN software, Adobe Photoshop, Image J software, and Imaris software (Bitplane).

### Terminal deoxynucleotidyltransferase-mediated dutp nick end labeling (TUNEL)

TUNEL staining was used to detect DNA fragmentation *in situ* and performed with the *In Situ* Cell Death Detection Kit, TMR red (Roche, Cat.-Nr. 12156792910) according to manufacturer's instructions. In brief, 40 μm brain sections of mouse brains were obtained as described above and blocked for 1 h at room temperature in TBS containing 0.5% Triton X-100, 0.1% Na-Azide, 0.1% Na-Citrate, and 5% normal goat serum. Sections were washed in PBS twice for 5 min each in PBS and incubated with the TUNEL labeling solution. Therefore, two brain sections were simultaneously incubated with 250 μl of TUNEL labeling solution in one well of a 24-well plate for 1 h at 37°C covered with aluminum foil. Sections were once washed with PBS containing Hoechst for 10 min at room temperature to stain nuclei. Before mounting sections in AquaMount (DAKO, Glostrup, Denmark) they were once washed in PBS for 10 min at room temperature. TUNEL positive (TUNEL+) cells were counted.

### Statistics

Graphics presented and statistics carried out were done using the statistical software “R” Version 2.15.0 (R Core Team, 2012). A significance-level (α) of 0.05 was selected. Data were analyzed using *t*-tests for comparisons between genotypes. The learning performance in the BM was analyzed using a repeated measures ANOVA with genotype as the between subject factor and trial as the repeated measure. Olfactory habituation within each genotype was analyzed by paired *t*-tests comparing the last trials of habituation with the respective first trial of a newly presented odor. Differences between genotypes were analyzed by unpaired *t*-tests comparing the first trials of each presented odor. In addition, Sigma Plot was used (Systat Software, Inc., San Jose, USA).

### Metabolite extraction

#### Brain tissue

For quenching, dissected brain tissues (mainly consisting of striatum, cortex, RMS, SVZ, and Hippocampus) were directly snap-frozen in liquid nitrogen and stored at −80°C until metabolite extraction. Pre-weighted brain tissues were transferred in 2 ml-Precellys tubes prefilled with 0.6 g ceramic beads (∅ 1.4 mm, Peqlab, Germany) and the appropriate amount of extraction fluid (MeOH/H2O, 40+8.5, v/v) was added. For sample lysis, a Precellys24 (Bertin, France) homogenizer was used (30 s at 6000 rpm). The temperature was held at 0°C by using the Cryolys cooling option (Bertin, France). Then water (200 μl/100 mg tissue) was added to the homogenized tissue fluid, followed by chloroform (400 μl/100 mg tissue). The homogenate was incubated for 20 min at 4°C under continuous shaking. After the incubation period, the samples were centrifuged at 14,000 ×g for 5 min at 4°C. Finally, 20 μl of the upper aqueous phase were transferred into a sample glass vial with micro insert. Samples were evaporated using a CentriVap Concentrator (Labconco, USA) at −4°C.

#### Cell culture

Cells were grown in six-well plates. For higher signal intensity two wells were pooled. First, the cells in all wells were washed with 1 ml 0.9% NaCl. After quenching with 0.4 ml cold methanol (−20°C) and adding an equal volume of cold water (4 °C), cells were collected with a cell scraper and transferred into the second well followed by cell scraping.

The cell extract was transferred into reaction tubes containing cold chloroform (−20°C). The extracts were incubated at 4°C for 20 min under shaking followed by centrifugation at 16,000 ×g for 5 min at 4°C. 0.3 ml of the polar phase were transferred into sample glass vials with micro inserts and evaporated using a CentriVap Concentrator (Labconco, USA) at −4°C.

### Derivatization and GC-MS analysis

Metabolite derivatization was performed by using a multi-purpose sampler (GERSTEL, Germany). Dried samples were dissolved in 15 μl pyridine, containing 20 mg/ml methoxyamine hydrochloride, at 40°C for 60 min under shaking. After adding 15 μl N-methyl-N-trimethylsilyl-triflouroacetamide (MSTFA) samples were incubated at 40°C for 30 min under continuous shaking.

GC-MS analysis was performed by using an Agilent 7890A GC coupled to an Agilent 5975C inert XL MSD (Agilent Technologies, Germany). A sample volume of 1 μl was injected into a Split/Splitless inlet operating in splitless mode at 270°C. The gas chromatograph was equipped with a 30 m DB-35MS capillary column + 5 m DuraGuard capillary in front of the analytical column (Agilent J&W GC Column).

#### Brain tissue extracts

Helium was used as carrier gas with a constant flow rate of 1.2 ml/min. The GC oven temperature was held at 80°C for 1 min and increased to 320°C at 15°C/min. The final temperature was held for 8 min. The total run time was 25 min.

#### Cell culture extracts

Helium was used as carrier gas with a constant flow rate of 1.0 ml/min. The GC oven temperature was held at 80°C for 6 min and increased to 300°C at 6°C/min. After 10 min, the temperature was increased at 10°C/min to 325°C for 4 min. The total run time was 59.167 min.

The transfer line temperature was set constantly to 280°C. The MSD was operating under electron ionization at 70 eV. The MS source was held at 230°C and the quadrupole at 150°C. Full scan mass spectra were acquired from m/z 70 to 800. The total run time was 25 min. Ion-chromatographic deconvolution, chromatogram alignment, identification and semi-quantification of metabolite amounts was done with the MetaboliteDetector software (Hiller et al., 2009). TIC normalization was performed to minimize systematic errors during measurement. In detail, the peak area of every compound in a sample was divided by the summed sample signal of all compounds in this sample.

### Metabolic network modeling

Metabolic network models for wild type and TRIM32 mutated neuronal stem cells were built with the FASTCORMICS workflow (Pacheco and Sauter, 2014) that allows the reconstruction of metabolic models based on microarray data. FASTCORMICS comprises a discretization step based on barcode (Zilliox and Irizarry, 2007), that computes for each probe set of the microarray a z-score for the measured intensity levels after frma normalization (McCall et al., 2010) against a standard intensity distribution of non-expressed genes obtained from a collection of thousands of arrays for the same platform stored in the mogene.1.0.st.v1frmavecs vector. The z-scores were then mapped to the reactions of the mouse model iSS1393 (Heinken et al., 2013) via the Gene-Protein Rules. Reactions with z-scores of zero and below in two out of three arrays were considered as non-expressed and removed from the model. Reactions associated to z-scores above 5 in two of three arrays constitute the set of core reactions. FASTCORMICS builds then consistent compact models that contain a maximal number of core reactions for the two different conditions while not including reactions regulated by non-expressed genes. Random sampling (Becker et al., 2007) of the possible solution space was then performed to obtain a qualitative estimate of the flux distribution allowed by the network topology and constraints of the two models when maximizing for the glycolysis pathway (PGM).

## Results

### TRIM32 is upregulated upon differentiation of subgranular and subventricular zone neural stem cells

To analyse TRIM32 protein expression by immunofluorescence stainings in NSCs of the adult brain, we used sections from Nestin-GFP mice. In these mice, NSCs in the SVZ and the DG express GFP driven by a Nestin promotor (Yamaguchi et al., 2000). Expression of TRIM32 protein in the SVZ-OB system has been described earlier (Hillje et al., 2013) and was repeatedly analyzed to compare expression levels of TRIM32 protein in progenitor cells of the SVZ with progenitor cells of the DG. Staining of sections from Nestin-GFP mice with an antibody against TRIM32, that has been shown to be specific before in immunohistochemical as well as biochemical approaches (Schwamborn et al., 2009; Hillje et al., 2011), revealed that TRIM32 protein is virtually absent from adult NSCs (type B cells) in the SVZ as well as DG NSCs (Figure 1A). NSCs and neuroblasts reside only the very inner layer of the DG. As soon as they become immature neurons and finally granule cells, they enter the outer layers of the DG. Cells in these layers show a strong nuclear TRIM32 expression (Figure 1A), indicating that TRIM32 protein is upregulated upon differentiation of DG NSCs.

Stem cells of the SVZ give rise to transient amplifying cells, which in turn differentiate into neuroblasts. Neuroblasts are generated in the SVZ and migrate toward the OB along the RMS. Co-staining of the neuroblast marker doublecortin and TRIM32 in sections from adult wildtype mice (wt) brains indicate that neuroblasts located in the distal part of the RMS express TRIM32 in the cytoplasm as well as in the nucleus (Figure 1B). Once these cells reach the OB, TRIM32 is strongly expressed in the nucleus. These data indicate that TRIM32 protein expression is upregulated upon neuronal differentiation of DG subgranular and SVZ NSCs. Furthermore, they are in good agreement with the TRIM32 expression pattern in the SVZ-OB system that we have shown previously (Hillje et al., 2013).

### Loss of TRIM32 leads to more newly generated neurons and less apoptosis in the SVZ OB system but not the DG

**Figure 2:**
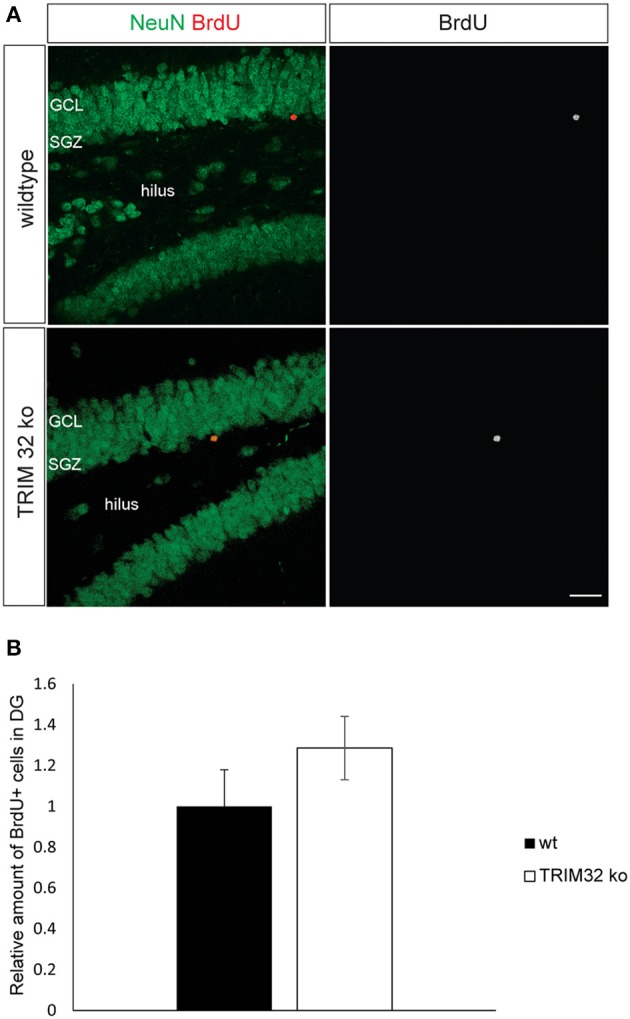
**Loss of TRIM32 is not influencing the rates of adult-born neurons in the dentate gyrus (DG) significantly. (A)** Freefloating sections including the DG of wildtype (wt) and TRIM32 ko mice that were injected with BrdU and stained with the indicated antibodies. Scale bar = 20 μm. **(B)** shows the quantifications of **(A)**. *N* = 5 mice (*p* < 0.05). GCL, granular cell layer; SGZ, subgranular zone.

Since TRIM32 is upregulated during the critical period of differentiation of progenitors into neurons in the SVZ and DG, we analyzed rates of neurogenesis in the SVZ – OB system and DG of wt and TRIM32 deficient mice. BrdU was applied to mice from both genotypes on 3 consecutive days and the brains were fixed 14 days after the last injection. Compared to wt mice, we found a significant increase in the density of BrdU+ cells in the granule cell layer (GC) of the OB of TRIM32 ko mice (Hillje et al., 2013). In contrast, also there is a tendency toward more BrdU+ cells in the DG of TRIM32 ko mice, this tendency did not reach statistical significance (Figures 2A,B). The higher density of BrdU+ cells in the OB GC of TRIM32 ko mice could either be due to an increase in proliferation of progenitor cells or increased survival of newly generated neurons in the OB. Recently, we have shown that the density of cell cycle active cells (Ki67+) is higher in the SVZ-OB system of TRIM32 ko mice (Hillje et al., 2013). However, the density of cell cycle active cells was unchanged in the DG (data not shown). Concerning the rate of cell death, the amount of Casp3+ as well as TUNEL+ cells was significantly reduced in the OB of TRIM32 deficient mice (Hillje et al., 2013). In the DG, we did not find significant changes in the amount of apoptotic cells (Supplementary Figure 1). Taken together, these data implicate that loss of TRIM32 leads to an overproduction of newly generated neurons and less apoptosis in the OB. No significant changes were observed in the DG.

### Loss of TRIM32 does not impair exploratory behavior, anxiety related behavior and spatial learning but leads to increased numbers of stops in the open field test

A health check did not reveal any significant differences between the genotypes. Furthermore, no severe deficits that would have excluded animals of either genotype from further analysis were observed. The weight development of individual mice was monitored during the course of the study for 15 weeks. At first weighing at an age of 22 days, TRIM32 knockout mice weighed significantly less compared with wild-type conspecifics (mean ko: 6.78 g, wt: 7.96 g). This difference disappeared during the following weeks where both genotypes were virtually indistinguishable from each other (Supplementary Figure 2).

**Figure 3:**
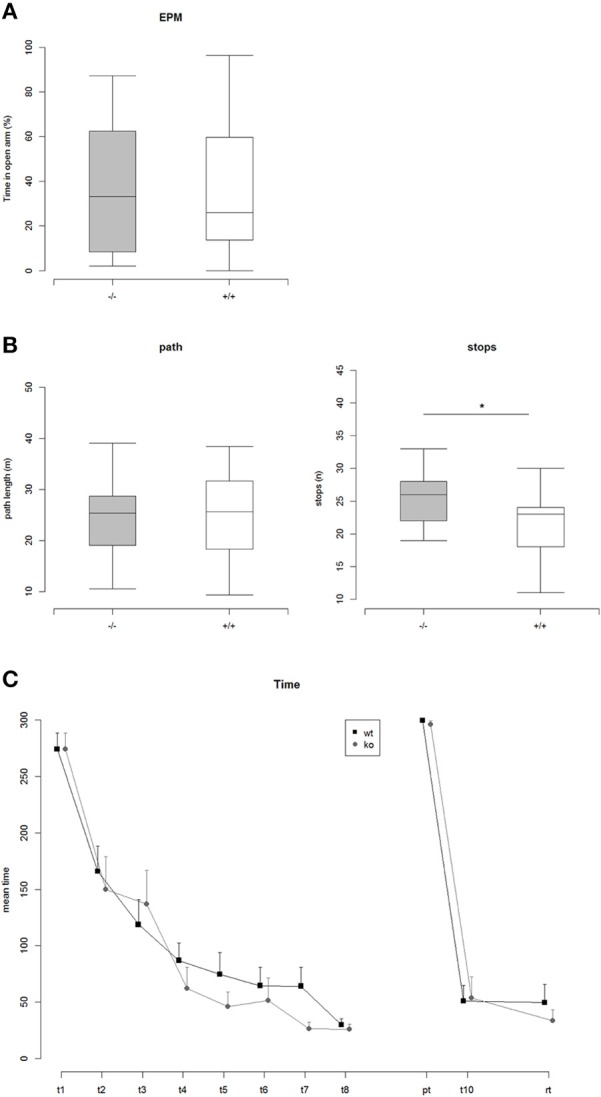
**(A)** Percent time on open arms in the Elevated Plus Maze (EPM): ko vs. wt: Two Sample *t*-test, *t* = 0.17, *p* = 0.86 (n.s.), Nko = 14, Nwt = 20. **(B)** Left: Path length traveled in the Open Field Test: ko vs. wt: Two Sample *t*-test, *t* = 0.15, *p* = 0.88 (n.s.), Nko = 14, Nwt = 21. Right: Number of stops while exploring the Open Field arena: ko vs. wt: Two Sample *t*-test, *t* = 2.36, *p* = 0.025 (^*^), Nko = 14, Nwt = 21. **(C)** Mean time to find the correct hole on the Barnes maze. Repeated measures ANOVA revealed a highly significant effect of trial, indicating that both genotypes learned the position of the correct hole [*F*_(1, 170)_ = 392.86, *p* < 2e-16]. There was no effect of genotype. In addition a comparison of the areas under the learning curves did not reveal any significant differences between ko vs. wt, AUC-Analysis: Two Sample *t*-test, *t* = 1.02, *p* = 0.32.

In the Elevated Plus Maze neither the percentage of time spent on open arms nor the percentage of entries into open arms did differ significantly between the genotypes (Figure 3A). Additionally measured parameters for general activity did not reveal any significant genotype effects. In the Open Field Test no significant differences between the genotypes were detectable concerning the traveled path length (Figure 3B). However, TRIM32 knockout mice were observed to show a significantly increased number of stops (Figure 3B).

In order to analyze visual spatial memory a Barnes Maze test was conducted. Mice of both genotypes significantly learned to find the position of the correct hole during the course of the training phase of 4 consecutive days indicated by a significant trial effect revealed by a repeated measures ANOVA [*F*_(1, 170)_ = 392.86, *p* < 2e-16] (Figure 3C). The genotypes, however, did differ neither during the training phase nor in the probe trial or the re-trial. Thus, loss of TRIM32 does not impair exploratory behavior, anxiety-related behavior but leads to increased number of stops in the open field test.

### TRIM32 deficiency impairs olfactory discrimination

**Figure 4:**
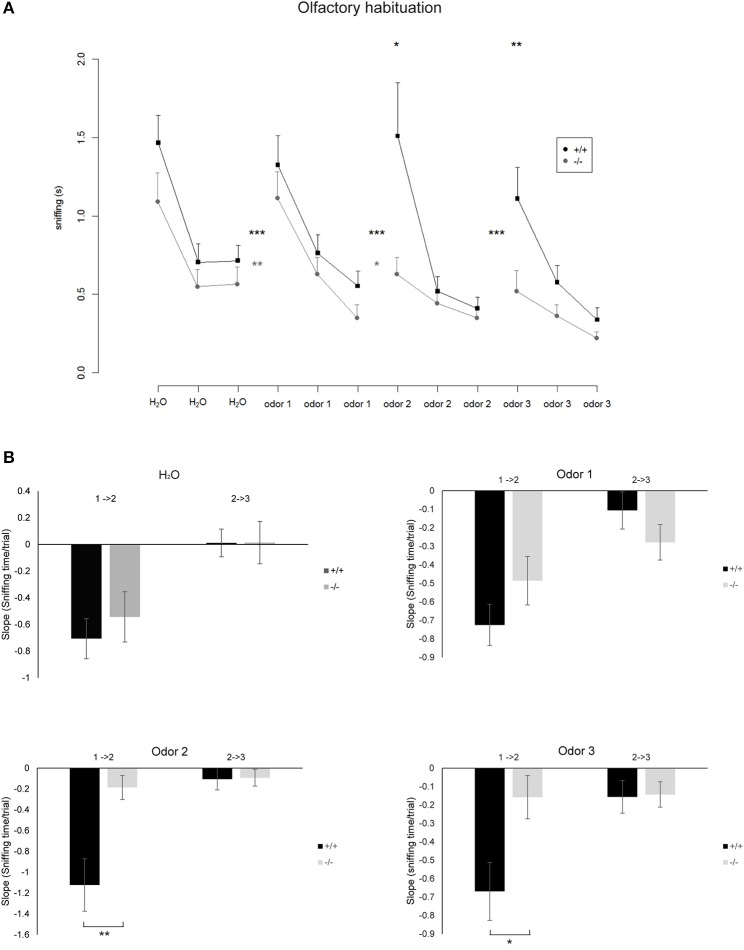
**(A)** Mean time spent sniffing on different odors in the Olfactory Habituation Test. Odors were presented three times in a row and subsequently a new odor was presented. Significant differences between the time spent sniffing in the last trial of a known odor compared with the first presentation of a new odor are indicated by asterisks between the lines (paired *t*-tests, ^*^p < 0.05, ^**^p < 0.01, ^***^p < 0.001). Differences between the genotypes regarding the time spent sniffing in the first trial of a newly presented odor are indicated by asterisks above the curves (Two sample unpaired *t*-tests). Nko = 14, Nwt = 18. **(B)** Bar diagrams representing the slope that indicates the rate at which the sniffing time decreases from the first to the second trial (1 → 2) and second to the third trial (2 → 3) for the indicated odors. Differences in genotypes are indicated by asterisks (^*^p < 0.05, ^**^p < 0.01) (according to normal distribution of the values *t*-test for odor 1, Mann–Whitney Rank Sum Test for odor 2 and 3). Nko = 14, Nwt = 18.

To determine the influence of increased neurogenesis due to loss of TRIM32 on olfactory capabilities, olfactory memory was tested by means of an olfactory habituation test. Each tested mouse was firstly exposed to distilled water and subsequently to three different odors. Each substance was applied three times in a row with an inter trial interval of 30 s. Both genotypes habituated to the repeated presentation of distilled water on a cotton swab. After habituation to distilled water mice of both genotypes significantly increased sniffing time toward the new odor indicating general olfactory abilities (Figure 4A). This was true for all three odors. Mice of both genotypes habituated to the odor comparable to the presentation of distilled water. But, the decrease of the sniffing time from the first to the second presentation was significantly lower for TRIM32 ko mice compared to wt mice for odor 2 and 3 (Figure 4B). Compared to the initial sniffing time of the first trial, TRIM32 knockout mice spend less time sniffing at trial 2 and 3 without recognizing the already known odor. Thus, habituation levels were weaker for TRIM32 knockout mice.

In general, compared to wt mice, TRIM32 ko mice spend shorter sniffing times at the cotton swap for all odors already from the very first presentation of the odor. Taken together, increased rates of neurogenesis due to loss of TRIM32 do not lead to an increased olfactory activity but in contrast even impair olfactory performance.

### Loss of TRIM32 leads to deregulated brain metabolism

**Figure 5:**
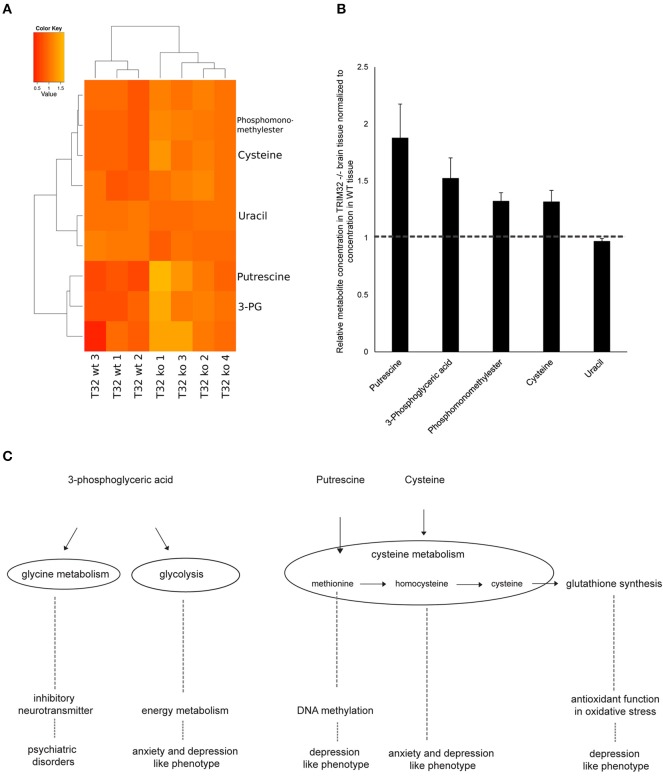
**(A)** Heatmap showing metabolites that differ significantly between wt and TRIM32 ko mice (Student's *t*-test, *p*-value < 0.05). Medians of three technical replicates were used as basis for this data analysis. For visualization the individual intensities for each compound were divided by its mean intensity across all replicates. Colors represent metabolite levels in TRIM32 ko and wt brain tissue. Clustering was performed on Euclidean distances using Ward's minimum variance method. Three wt and 4 ko animals were used for analysis. **(B)** Relative concentration of metabolites that differ significantly in concentration between wt and TRIM32 ko mice. Data were calculated as means with standard error of the mean and values of TRIM32 ko mice were normalized to wt values. **(C)** Schematic overview depicting metabolomic pathways to which metabolites that were significantly different in concentration are linked to and their involvement in brain disorders and behavioral phenotypes.

Accumulating evidence suggests that a deregulation of certain molecular pathways leads to the formation of brain disorders as well as anxiety and depression related phenotypes. To identify affected anxiety and depression related pathways, we performed metabolomic analyses from brain tissue of 3 wt and 4 TRIM32 ko mice. Two hundred and ten metabolites were detected by GC/MS of which 75 have been identified using our in-house mass spectral metabolite library (Supplementary Figure 3). Statistical analysis revealed that levels of nine out of these 210 metabolites differed significantly in levels of concentration between the two genotypes (*p* < 0.05). Of those, five have been be identified (Figure 5A). These metabolites are phosphomonomethylester, 3-phosphoglyceric acid, cysteine, putrescine, and uracil. The concentration of 3-phosphoglyceric acid, which is a metabolic intermediate in glycolysis and is also a switching point to glycine and serine metabolism, was significantly higher in the tissue of TRIM32 ko mice (Figures 5B,C). A similar significant increase was found for cysteine, which is related to serine, methionine, and glutathione metabolism. Concentrations of the degradation product putrescine, that has been linked to methionine metabolism as well, were elevated in a similar way (Figures 5B,C). The mouse brain tissue that was used for our metabolomics analysis not only contains NSCs but represents a mixture of multiple cell types. To investigate the relevance of these results for NSCs we performed the same analysis using a pure population of cultured NSCs (Supplementary Figure 4). From the five identified metabolites that significantly differed in their concentration from wt to TRIM32 ko brain tissue, putrescine and 3-phosphoglyceric acid were also significantly upregulated in TRIM32 ko NSCs.

**Figure 6:**
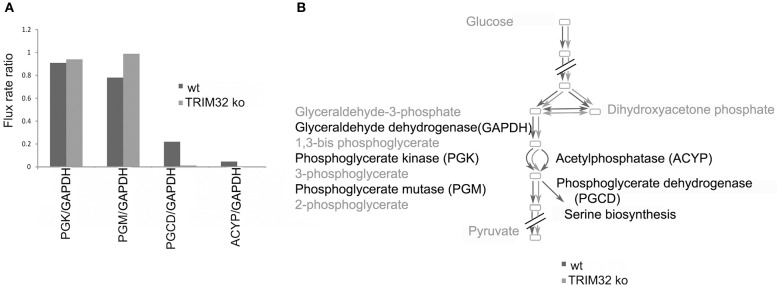
**Flux distribution estimated by random sampling for the wild type and the mutant models built via the FASTCORMICS workflow. (A)** Random sampling results. Ratio of the flux rates for phosphoglycerate kinase (PGK), phosphoglycerate mutase (PGM), and acetylphosphatase (ACYP) over glyceraldehyde dehydrogenase (GADP) represented in blue for the wild type and in red for the mutant. **(B)** Schematic representation of the qualitative wild type (dark gray) and mutant (light gray) fluxes over the glycolysis pathway.

Finally, we wanted to get a more systemic view of deregulated pathways that might lead to pathological changes in the brains of TRIM32 ko mice. Therefore, previously generated gene-expression data from TRIM32 ko mice (Hillje et al., 2013) were linked to the results that were obtained in the metabolomic analysis of wt and TRIM32 ko brains. Genome-scale metabolic network models were reconstructed via the FASTCORMICS workflow making use of the available microarray data (Pacheco and Sauter, 2014, arXiv:1407.6534). The wt and TRIM32 ko models contain 735 and 635 reactions, respectively, with 516 reactions being shared between the two models, indicating some metabolic differences between the two conditions. Both models are available in SBML format as Supplementary Files. A qualitative estimate of the flux distribution of the pathways containing the significantly changed metabolites was then obtained by random sampling of the possible solution space (Figure 6). It suggests in the ko model a decreased consumption of 3-phosphoglyceric acid by the phosphoglycerate dehydrogenase, the first enzyme in the serine biosynthesis pathway. This in turn leads to a slight accumulation of the glycolytic intermediate 3-phosphoglyceric acid, as observed in the metabolite measurements (Figure 5). Taken together, we were able to show that there is a difference in metabolic profiles between wt and TRIM32 ko brains. In addition, modeling the estimation of flux distribution suggests that the increase of 3-phosphoglyceric acid, which was significantly deregulated in the TRIM32 ko brain tissue as well as in the TRIM32 ko NSC culture, might be due to a decreased consumption by phosphoglycerate dehydrogenase.

## Discussion

We recently demonstrated that the absence of TRIM32 in knockout mice led to increased progenitor cell proliferation and less cell death, both effects accumulate in an overproduction of adult generated olfactory OB neurons of TRIM32 knockout mice (Hillje et al., 2013). Here, we show that such an increase does not necessarily lead to better olfactory performance but contrary, TRIM32 knockout mice even show impaired olfactory habituation.

The performed olfactory habituation assay evaluates the habituation to known odors as well as the detection of a new odor. Although it might be possible that TRIM32 ko mice merely lack interest or motivation in sniffing new odors, we believe that the increase of sniffing time for the first presentation of a new odor following the presentation of water or following the third presentation of an already presented odor indicates that there is general interest in a new odor. For both genotypes the sniffing time for the first presentation of odor 1 is significantly longer compared to the sniffing time of the last presentation of water. The same is true for the first presentation of odor 2. For the first presentation of odor 3 there is an increase even though not significant for TRIM32 ko mice. If TRIM32 ko mice would have no interest or motivation we would not expect to see this increase. In addition, these results point to the fact that TRIM32 ko mice are able to distinguish between the already known odor and a new odor. However, the lower decrease of sniffing time upon the second and third presentation of subsequently presented odors points to the fact that it takes TRIM32 ko mice longer to recognize the already known odor, meaning an impairment of olfactory memory and habituation. However, we cannot rule out that the lower decrease of sniffing time is a combinatorial effect of impaired memory (habituation), lower discrimination (olfactory capabilities), and lower interest. Anyways, since we tested a battery of non-olfactory based behavioral tests that showed no differences regarding emotional and motivational states (with respect to anxiety-related behavior tested in the EPM, motivation to gain access to the home cage and general learning performance tested in the Barnes Maze Test) we believe that lack of motivation is most probably not the main cause of the observed behavior. Furthermore, we also cannot fully exclude that TRIM32 deficient olfactory neurons are dysfunctional and this potential dysfunction contributes to the observed phenotypes.

Although there have been conflicting results in the past and its exact function could not be fully determined yet, adult neurogenesis seems to play an important role in olfactory processes including olfactory discrimination, memory and associative learning (for review see Breton-Provencher and Saghatelyan, 2012). Our data indicate that olfactory habituation was significantly impaired in TRIM32 ko mice. Thus, the mere number of newly generated neurons itself does not guarantee an improvement of olfactory information processing. These results are in line with findings by Mechawar et al. (2004), who found that an increased number of granule cells due to a decreased apoptosis rate did not result in enhanced olfactory abilities, but lead to a declined short-term memory of odors. We hypothesize that due to the increased proliferation and decreased cell removal rates, the newborn interneurons are defectively integrated into the circuitry and therefore the animals show impaired olfactory functioning. Strikingly, long-term memory seemed not to be affected of this as the knockout mice showed similar habituation to the odors as their conspecifics after a second introduction of the same odor set after 1 week (data not shown). Most interestingly, other behavioral domains (such as exploratory behavior, anxiety-like behavior, and spatial learning) were not affected by the lack of TRIM32. The only remarkable difference we found was the number of stops conducted during the Open Field Test. Stopping while exploring a new environment is a typical behavior shown by mice. We speculate that the later shown olfactory deficits most likely have increased the demand for such back-pedaling behavior in order to gain information in the face of impaired olfactory capacities.

There is growing evidence that deregulation in metabolite concentrations leads to the formation of brain disorders as well as anxiety- and depression-related phenotypes. Our statement that the cell fate determinant TRIM32 is required for a balanced activity of the adult neurogenesis process is supported by hints that point to a deregulation of several metabolic intermediates that are connected to glycolysis, glycine or cysteine metabolism in TRIM32 knockout mice brain tissue. Our data implicate that loss of TRIM32 leads to changes in the concentration of 3-phosphoglyceric acid, a metabolite linked to glycolysis. Rates of glycolysis have been shown to be deregulated in anxiety and depression-like phenotypes in systems biology approaches (Gormanns et al., 2011) as well as in different anxiety mouse models (Filiou et al., 2011; Zhang et al., 2011). In addition, our data show significant changes in the levels of cysteine due to loss of TRIM32. Cysteine is an intermediate of the trans-sulfuration pathway (methionine → homocysteine → cysteine) and thus glutathione synthesis. Glutathione is the major antioxidant of the brain and is of particular importance for defense mechanisms against oxidative damage. Methylation of DNA has been suggested to be involved in the cause of mood disorders and strongly relies on the availability of methyl groups from s-adenosyl methionine (SAM). After providing its methyl group, SAM is regenerated via homocysteine and methionine where the methyl group is provided either by trimethylglycine (betaine) or by 5-methyltetrahydrofolate. The latter mainly derives its methyl group from serine via 5,10-methylenetetrahydrofolate. Oxidative stress mechanisms and methylation have been implicated in remitted phases of major depressive disorders in humans (Kaddurah-Daouk et al., 2012) and deregulation in cysteine and methionine metabolism were linked to depression and anxiety in a systems biology approach as well as in a mouse model (Gormanns et al., 2011; Zhang et al., 2011). In addition to the above mentioned pathways, 3-phosphoglyceric acid functions in glycine and serine as well as cysteine metabolism. Glycine is an inhibitory neurotransmitter in the spinal cord and brain stem with a regulatory function in locomotor behavior (reviewed in Legendre, 2001; Xu and Gong, 2010). Glycine synaptic transmission was suggested to be involved in psychiatric disorders (Zhang et al., 2011). Since the used brain tissue contains multiple cell types, the metabolomics analysis was repeated using pure populations of cultured wt and TRIM32 ko NSCs. From the five identified metabolites that significantly differed in their concentration between the two genotypes in brain tissue, putrescine and 3-phosphoglyceric acid could be verified to be significantly differently abundant in wt and TRIM32 ko NSCs.

However, the metabolomics approach is limited by only taking a snapshot look at one static point. In order to get a more detailed understanding of metabolomics changes in TRIM32 ko brain, we modeled metabolomics fluxes by combining previously published gene expression (Hillje et al., 2013) and metabolic data. The ko model suggests a decreased consumption of 3-phosphoglycerate by the phosphoglycerate dehydrogenase, the first enzyme in the serine biosynthesis pathway, which leads to a slight accumulation of phosphoglycerate, as observed in the metabolite measurements of the brain tissue as well as the NSC cultures. The deficiency of 3-phosphoglycerate dehydrogenase (3-PGDH) is the most reported defect that causes serine deficiency disorders, a group of neurodevelopmental, neurometabolic disorders with congenital microcephaly, intractable seizures and severe psychomotor retardation (van der Crabben et al., 2013), implicating the pathological relevance of this pathway. In addition to deregulated metabolic fluxes, loss of TRIM32 results in an overproduction of adult generated OB neurons, and it cannot be excluded that different cell population sizes might cause changes of metabolite levels in TRIM32 ko brains.

Even though no clear anxiety or depression like phenotype was found for TRIM32 knockout mice in the behavioral tests, shorter sniffing times in olfactory habituation tests as well as more stops in the Open Field tests might be hints for lower motivation or might even indicate slight depression like behavior. However, the function of TRIM32 in depression and anxiety is still controversial. Using a chronic unpredictable mild stress (CUMS) mouse model that generates anxiety- and depression-like behavior, Ruan and colleagues showed that TRIM32 protein levels are downregulated in the hippocampus under mild stress (Ruan et al., 2014). However, at the same time they demonstrate that a total loss of TRIM32 (knock-out mouse model) protects against depression. Depression in general is associated to reduced levels of neurogenesis, while in TRIM32 knock-out mice neurogenesis is increased. It seems tempting to speculate that these two effects, in the CUMS depression model in TRIM32 knock-out mice, balance each other leading to a normalized neurogenesis activity. Although in our TRIM32 ko mice we did not observe any anxiety related phenotypes, in our metabolomics approach we detected several metabolites to be deregulated which previously have been shown to be implicated in anxiety and depression. Hence, such a systematic omics approach might be even more sensitive in revealing fine changes in complex behaviors, which might be missed by conventional behavioral assays.

Our study provides comprehensive data on how the deregulation of adult neurogenesis caused by the loss of the cell fate determinant TRIM32 leads to a deregulation of metabolomic pathways and finally results in an impairment of olfactory capabilities. These results highlight that the function of the cell fate-determinant TRIM32 for a balanced activity of the adult neurogenesis process exceeds the cellular level and has far-reaching effects on metabolomics pathways that are linked to mood disorders as well as olfactory capabilities.

## Author Contributions

AH, EB, JCS, and LL designed the study. Behavioral experiments were conducted by EB and LL; Immunofluorescence staining based experiments were conducted by AH, EB, MASP, and JCS; Metabolomics experiments and metabolic modeling were done by CJ, MPP, and TS. All authors contributed to the analysis of obtained data. AH, EB, JCS, LL wrote the manuscript, while MASP, CJ, MPP, and TS edited the manuscript.

### Conflict of interest statement

The authors declare that the research was conducted in the absence of any commercial or financial relationships that could be construed as a potential conflict of interest.

## Supplementary material

Supplementary Figure 1 **(A)** Free floating sections from wt and TRIM32 ko mouse brain stained with TUNEL and Hoechst. Shown is the dentate gyrus of the hippocampus. Scale bar = 20 μm. **(B)** Quantification of TUNEL+ cells in dentate gyrus of wt and TRIM32 ko mouse brain. *N* = 4 wt and 4 TRIM32 ko mice, *n* = 23 cells.

Supplementary Figure 2 **Weight development over the course of 15 weeks**. Two Sample *t*-test, *t* = 2.743, *df* = 53, *p*-value = 0.0083.

Supplementary Figure 3 **Heatmap showing relative levels of metabolites in wt and TRIM32 ko mice**. Data were calculated as medians. Colors represent relative changes of concentration in wt and TRIM32 ko brain tissue. Unlabeled rows represent unknown metabolites. Metabolome clusters consist of samples with similar concentration profiles. Three wt and 4 ko animals were used for analysis.

Supplementary Figure 4 **(A)** Hierarchical cluster analysis of significantly changed metabolite levels in NSCs (wild type vs. TRIM32 knock-out). The statistical significance and robustness of differences in metabolite levels (Welch's *t*-test, *p* < 0.05, *n* = 6) was evaluated by leave-one-out cross-validation. **(B)** Bar diagram showing significantly changed metabolite levels in NSCs (wild type vs. TRIM32 ko). Statistical analysis see **(A)**, data were calculated as means with standard error of the mean and values of TRIM32 ko mice were normalized to wt values.

Supplementary Files **Genome-scale metabolic network models**. Zipped file containing the SBML versions of the reconstructed genome scale metabolic network models of the wild type and the TRIM32 ko brain tissue.

## References

[B2] Bardy C. Alonso M. Bouthour W. Lledo P. M. (2010). How, when, and where new inhibitory neurons release neurotransmitters in the adult olfactory bulb. J. Neurosci. 30, 17023–17034. 10.1523/JNEUROSCI.4543-10.2010 21159972 PMC6634902

[B3] Barnes C. A. (1979). Memory deficits associated with senescence: a neurophysiological and behavioral study in the rat. J. Comp. Physiol. Psychol. 93, 74–104. 10.1037/h0077579 221551

[B4] Becker S. A. Feist A. M. Mo M. L. Hannum G. Palsson B. O. Herrgard M. J. (2007). Quantitative prediction of cellular metabolism with constraint-based models: the COBRA Toolbox. Nat. Protoc. 2, 727–738. 10.1038/nprot.2007.99 17406635

[B5] Bello B. Reichert H. Hirth F. (2006). The brain tumor gene negatively regulates neural progenitor cell proliferation in the larval central brain of Drosophila. Development (Cambridge, England) 133, 2639–2648. 10.1242/dev.02429 16774999

[B6] Belluzzi O. Benedusi M. Ackman J. LoTurco J. J. (2003). Electrophysiological differentiation of new neurons in the olfactory bulb. J. Neurosci. 23, 10411–10418. Available online at: http://www.jneurosci.org/content/23/32/10411.full 14614100 10.1523/JNEUROSCI.23-32-10411.2003 PMC6741027

[B7] Betschinger J. Knoblich J. A. (2004). Dare to be different: asymmetric cell division in Drosophila, *C. elegans* and vertebrates. Curr. Biol. 14, R674–685. 10.1016/j.cub.2004.08.017 15324689

[B8] Breton-Provencher V. Saghatelyan A. (2012). Newborn neurons in the adult olfactory bulb: unique properties for specific odor behavior. Behav. Brain Res. 227, 480–489. 10.1016/j.bbr.2011.08.001 21843557

[B9] Carleton A. Petreanu L. T. Lansford R. Alvarez-Buylla A. Lledo P. M. (2003). Becoming a new neuron in the adult olfactory bulb. Nat. Neurosci. 6, 507–518. 10.1038/nn1048 12704391

[B10] Chiang A. P. Beck J. S. Yen H. J. Tayeh M. K. Scheetz T. E. Swiderski R. E.. (2006). Homozygosity mapping with SNP arrays identifies TRIM32, an E3 ubiquitin ligase, as a Bardet-Biedl syndrome gene (BBS11). Proc. Natl. Acad. Sci. U.S.A. 103, 6287–6292. 10.1073/pnas.0600158103 16606853 PMC1458870

[B11] Doetsch F. Caille I. Lim D. A. Garcia-Verdugo J. M. Alvarez-Buylla A. (1999). Subventricular zone astrocytes are neural stem cells in the adult mammalian brain. Cell 97, 703–716. 10.1016/S0092-8674(00)80783-7 10380923

[B12] Doetsch F. Garcia-Verdugo J. M. Alvarez-Buylla A. (1997). Cellular composition and three-dimensional organization of the subventricular germinal zone in the adult mammalian brain. J. Neurosci. 17, 5046–5061. 9185542 10.1523/JNEUROSCI.17-13-05046.1997 PMC6573289

[B13] Ernst A. Alkass K. Bernard S. Salehpour M. Perl S. Tisdale J.. (2014). Neurogenesis in the striatum of the adult human brain. Cell 156, 1072–1083. 10.1016/j.cell.2014.01.044 24561062

[B14] Filiou M. D. Zhang Y. Teplytska L. Reckow S. Gormanns P. Maccarrone G.. (2011). Proteomics and metabolomics analysis of a trait anxiety mouse model reveals divergent mitochondrial pathways. Biol. Psychiatry 70, 1074–1082. 10.1016/j.biopsych.2011.06.009 21791337

[B15] Frosk P. Greenberg C. R. Tennese A. A. Lamont R. Nylen E. Hirst C.. (2005). The most common mutation in FKRP causing limb girdle muscular dystrophy type 2I (LGMD2I) may have occurred only once and is present in Hutterites and other populations. Hum. Mutat. 25, 38–44. 10.1002/humu.20110 15580560

[B16] Frosk P. Weiler T. Nylen E. Sudha T. Greenberg C. R. Morgan K.. (2002). Limb-girdle muscular dystrophy type 2H associated with mutation in TRIM32, a putative E3-ubiquitin-ligase gene. Am. J. Hum. Genet. 70, 663–672. 10.1086/339083 11822024 PMC447621

[B17] Gage F. H. (2000). Mammalian neural stem cells. Science (New York, N.Y.) 287, 1433–1438. 10.1126/science.287.5457.1433 10688783

[B18] Gormanns P. Mueller N. S. Ditzen C. Wolf S. Holsboer F. Turck C. W. (2011). Phenome-transcriptome correlation unravels anxiety and depression related pathways. J. Psychiatr. Res. 45, 973–979. 10.1016/j.jpsychires.2010.12.010 21255794

[B19] Hammell C. M. Lubin I. Boag P. R. Blackwell T. K. Ambros V. (2009). nhl-2 Modulates microRNA activity in *Caenorhabditis elegans*. Cell 136, 926–938. 10.1016/j.cell.2009.01.053 19269369 PMC2670343

[B20] Heinken A. Sahoo S. Fleming R. M. Thiele I. (2013). Systems-level characterization of a host-microbe metabolic symbiosis in the mammalian gut. Gut Microbes 4, 28–40. 10.4161/gmic.22370 23022739 PMC3555882

[B21] Hiller K. Hangebrauk J. Jager C. Spura J. Schreiber K. Schomburg D. (2009). MetaboliteDetector: comprehensive analysis tool for targeted and nontargeted GC/MS based metabolome analysis. Anal. Chem. 81, 3429–3439. 10.1021/ac802689c 19358599

[B22] Hillje A. L. Pavlou M. A. Beckmann E. Worlitzer M. M. Bahnassawy L. Lewejohann L.. (2013). TRIM32-dependent transcription in adult neural progenitor cells regulates neuronal differentiation. Cell Death Dis. 4, e976. 10.1038/cddis.2013.487 24357807 PMC3877558

[B23] Hillje A. L. Worlitzer M. M. Palm T. Schwamborn J. C. (2011). Neural stem cells maintain their stemness through protein kinase C zeta-mediated inhibition of TRIM32. Stem Cells (Dayton, Ohio) 29, 1437–1447. 10.1002/stem.687 21732497

[B24] Horn E. J. Albor A. Liu Y. El-Hizawi S. Vanderbeek G. E. Babcock M.. (2004). RING protein Trim32 associated with skin carcinogenesis has anti-apoptotic and E3-ubiquitin ligase properties. Carcinogenesis 25, 157–167. 10.1093/carcin/bgh003 14578165

[B25] Kaddurah-Daouk R. Yuan P. Boyle S. H. Matson W. Wang Z. Zeng Z. B.. (2012). Cerebrospinal fluid metabolome in mood disorders-remission state has a unique metabolic profile. Sci. Rep. 2:667. 10.1038/srep00667 22993692 PMC3446657

[B26] Kano S. Miyajima N. Fukuda S. Hatakeyama S. (2008). Tripartite motif protein 32 facilitates cell growth and migration via degradation of Abl-interactor 2. Cancer Res. 68, 5572–5580. 10.1158/0008-5472.CAN-07-6231 18632609

[B27] Kudryashova E. Kramerova I. Spencer M. J. (2012). Satellite cell senescence underlies myopathy in a mouse model of limb-girdle muscular dystrophy 2H. J. Clin. Invest. 122, 1764–1776. 10.1172/JCI59581 22505452 PMC3336976

[B28] Kudryashova E. Kudryashov D. Kramerova I. Spencer M. J. (2005). Trim32 is a ubiquitin ligase mutated in limb girdle muscular dystrophy type 2H that binds to skeletal muscle myosin and ubiquitinates actin. J. Mol. Biol. 354, 413–424. 10.1016/j.jmb.2005.09.068 16243356

[B29] Kudryashova E. Wu J. Havton L. A. Spencer M. J. (2009). Deficiency of the E3 ubiquitin ligase TRIM32 in mice leads to a myopathy with a neurogenic component. Hum. Mol. Genet. 18, 1353–1367. 10.1093/hmg/ddp036 19155210 PMC2722196

[B30] Kuhn H. G. Dickinson-Anson H. Gage F. H. (1996). Neurogenesis in the dentate gyrus of the adult rat: age-related decrease of neuronal progenitor proliferation. J. Neurosci. 16, 2027–2033. 8604047 10.1523/JNEUROSCI.16-06-02027.1996 PMC6578509

[B31] Lazarini F. Lledo P. M. (2011). Is adult neurogenesis essential for olfaction? Trends Neurosci. 34, 20–30. 10.1016/j.tins.2010.09.006 20980064

[B32] Lee C. Y. Robinson K. J. Doe C. Q. (2006). Lgl, Pins and aPKC regulate neuroblast self-renewal versus differentiation. Nature 439, 594–598. 10.1038/nature04299 16357871

[B33] Legendre P. (2001). The glycinergic inhibitory synapse. Cell. Mol. Life Sci. 58, 760–793. 10.1007/PL00000899 11437237 PMC11337367

[B34] Lewejohann L. Skryabin B. V. Sachser N. Prehn C. Heiduschka P. Thanos S.. (2004). Role of a neuronal small non-messenger RNA: behavioural alterations in BC1 RNA-deleted mice. Behav. Brain Res. 154, 273–289. 10.1016/j.bbr.2004.02.015 15302134

[B35] Lionel A. C. Crosbie J. Barbosa N. Goodale T. Thiruvahindrapuram B. Rickaby J.. (2011). Rare copy number variation discovery and cross-disorder comparisons identify risk genes for ADHD. Sci. Transl. Med. 3, 95ra75. 10.1126/scitranslmed.3002464 21832240

[B36] Lionel A. C. Tammimies K. Vaags A. K. Rosenfeld J. A. Ahn J. W. Merico D.. (2014). Disruption of the ASTN2/TRIM32 locus at 9q33.1 is a risk factor in males for autism spectrum disorders, ADHD and other neurodevelopmental phenotypes. Hum. Mol. Genet. 23, 2752–2768. 10.1093/hmg/ddt669 24381304 PMC3990173

[B37] Mandairon N. Sacquet J. Garcia S. Ravel N. Jourdan F. Didier A. (2006). Neurogenic correlates of an olfactory discrimination task in the adult olfactory bulb. Eur. J. Neurosci. 24, 3578–3588. 10.1111/j.1460-9568.2006.05235.x 17229106

[B38] McCall M. N. Bolstad B. M. Irizarry R. A. (2010). Frozen robust multiarray analysis (fRMA). Biostatistics 11, 242–253. 10.1093/biostatistics/kxp059 20097884 PMC2830579

[B39] Mechawar N. Saghatelyan A. Grailhe R. Scoriels L. Gheusi G. Gabellec M. M.. (2004). Nicotinic receptors regulate the survival of newborn neurons in the adult olfactory bulb. Proc. Natl. Acad. Sci. U.S.A. 101, 9822–9826. 10.1073/pnas.0403361101 15210938 PMC470758

[B40] Ming G. L. Song H. (2011). Adult neurogenesis in the mammalian brain: significant answers and significant questions. Neuron 70, 687–702. 10.1016/j.neuron.2011.05.001 21609825 PMC3106107

[B41] Nicklas S. Otto A. Wu X. Miller P. Stelzer S. Wen Y.. (2012). TRIM32 regulates skeletal muscle stem cell differentiation and is necessary for normal adult muscle regeneration. PLoS ONE 7:e30445. 10.1371/journal.pone.0030445 22299041 PMC3267731

[B42] Nissant A. Bardy C. Katagiri H. Murray K. Lledo P. M. (2009). Adult neurogenesis promotes synaptic plasticity in the olfactory bulb. Nat. Neurosci. 12, 728–730. 10.1038/nn.2298 19412168

[B43] Nissant A. Pallotto M. (2011). Integration and maturation of newborn neurons in the adult olfactory bulb–from synapses to function. Eur. J. Neurosci. 33, 1069–1077. 10.1111/j.1460-9568.2011.07605.x 21395850

[B44] Pacheco M. P. Sauter T. (2014). Fast reconstruction of compact context-specific metabolic networks via integration of microarray data. arXiv:1407.6534

[B45] Petreanu L. Alvarez-Buylla A. (2002). Maturation and death of adult-born olfactory bulb granule neurons: role of olfaction. J. Neurosci. 22, 6106–6113 Available online at: http://www.jneurosci.org/content/22/14/6106.long 12122071 10.1523/JNEUROSCI.22-14-06106.2002 PMC6757952

[B45a] R Core Team. (2012). R: A Language and Environment for Statistical Computing. Vienna: R Foundation for Statistical Computing. Available online at: http://www.R-project.org/

[B46] Ruan C. S. Wang S. F. Shen Y. J. Guo Y. Yang C. R. Zhou F. H.. (2014). Deletion of TRIM32 protects mice from anxiety- and depression-like behaviors under mild stress. Eur. J. Neurosci. 40, 2680–2690. 10.1111/ejn.12618 24839933

[B47] Schwamborn J. C. Berezikov E. Knoblich J. A. (2009). The TRIM-NHL protein TRIM32 activates microRNAs and prevents self-renewal in mouse neural progenitors. Cell 136, 913–925. 10.1016/j.cell.2008.12.024 19269368 PMC2988196

[B48] Stuchlik A. (2014). Dynamic learning and memory, synaptic plasticity and neurogenesis: an update. Front. Behav. Neurosci. 8:106. 10.3389/fnbeh.2014.00106 24744707 PMC3978286

[B49] Tan J. Savigner A. Ma M. Luo M. (2010). Odor information processing by the olfactory bulb analyzed in gene-targeted mice. Neuron 65, 912–926. 10.1016/j.neuron.2010.02.011 20346765 PMC2901914

[B50] Toni N. Teng E. M. Bushong E. A. Aimone J. B. Zhao C. Consiglio A.. (2007). Synapse formation on neurons born in the adult hippocampus. Nat. Neurosci. 10, 727–734. 10.1038/nn1908 17486101

[B51] Urban N. N. (2002). Lateral inhibition in the olfactory bulb and in olfaction. Physiol. Behav. 77, 607–612. 10.1016/S0031-9384(02)00895-8 12527007

[B52] van der Crabben S. N. Verhoeven-Duif N. M. Brilstra E. H. Van Maldergem L. Coskun T. Rubio-Gozalbo E.. (2013). An update on serine deficiency disorders. J. Inherit. Metab. Dis. 36, 613–619. 10.1007/s10545-013-9592-4 23463425

[B53] van Praag H. Schinder A. F. Christie B. R. Toni N. Palmer T. D. Gage F. H. (2002). Functional neurogenesis in the adult hippocampus. Nature 415, 1030–1034. 10.1038/4151030a 11875571 PMC9284568

[B54] Vivar C. van Praag H. (2013). Functional circuits of new neurons in the dentate gyrus. Front. Neural Circuits 7:15. 10.3389/fncir.2013.00015 PMC3580993 23443839

[B55] Xu T. L. Gong N. (2010). Glycine and glycine receptor signaling in hippocampal neurons: diversity, function and regulation. Prog. Neurobiol. 91, 349–361. 10.1016/j.pneurobio.2010.04.008 20438799

[B55a] Yamaguchi M. Saito H. Suzuki M. Mori K. (2000). Visualization of neurogenesis in the central nervous system using nestin promoter-GFP transgenic mice. Neuroreport 11, 1991–1996. 10.1097/00001756-200006260-00037 10884058

[B56] Yang M. Crawley J. N. (2009). Simple behavioral assessment of mouse olfaction. Curr. Protoc. Neurosci. Chapter 8, Unit 8.24. 10.1002/0471142301.ns0824s48 PMC2753229 19575474

[B57] Yao J. Mu Y. Gage F. H. (2012). Neural stem cells: mechanisms and modeling. Protein Cell 3, 251–261. 10.1007/s13238-012-2033-6 22549585 PMC4875476

[B58] Yokoi M. Mori K. Nakanishi S. (1995). Refinement of odor molecule tuning by dendrodendritic synaptic inhibition in the olfactory bulb. Proc. Natl. Acad. Sci. U.S.A. 92, 3371–3375. 10.1073/pnas.92.8.3371 7724568 PMC42168

[B59] Yokota T. Mishra M. Akatsu H. Tani Y. Miyauchi T. Yamamoto T.. (2006). Brain site-specific gene expression analysis in Alzheimer's disease patients. Eur. J. Clin. Invest. 36, 820–830. 10.1111/j.1365-2362.2006.01722.x 17032350

[B60] Zhang Y. Filiou M. D. Reckow S. Gormanns P. Maccarrone G. Kessler M. S.. (2011). Proteomic and metabolomic profiling of a trait anxiety mouse model implicate affected pathways. Mol. Cell. Proteomics 10, M111.008110. 10.1074/mcp.M111.008110 21862759 PMC3237072

[B61] Zhao C. Deng W. Gage F. H. (2008). Mechanisms and functional implications of adult neurogenesis. Cell 132, 645–660. 10.1016/j.cell.2008.01.033 18295581

[B62] Zilliox M. J. Irizarry R. A. (2007). A gene expression bar code for microarray data. Nat. Methods 4, 911–913. 10.1038/nmeth1102 17906632 PMC3154617

