# Supplementary figures and images for: The neural stem cell fate determinant TRIM32 regulates complex behavioral traits
Source: Front Cell Neurosci. 2015 Mar 18;9:75. doi: 10.3389/fncel.2015.00075 (PMC4364253; doi:10.3389/fncel.2015.00075)

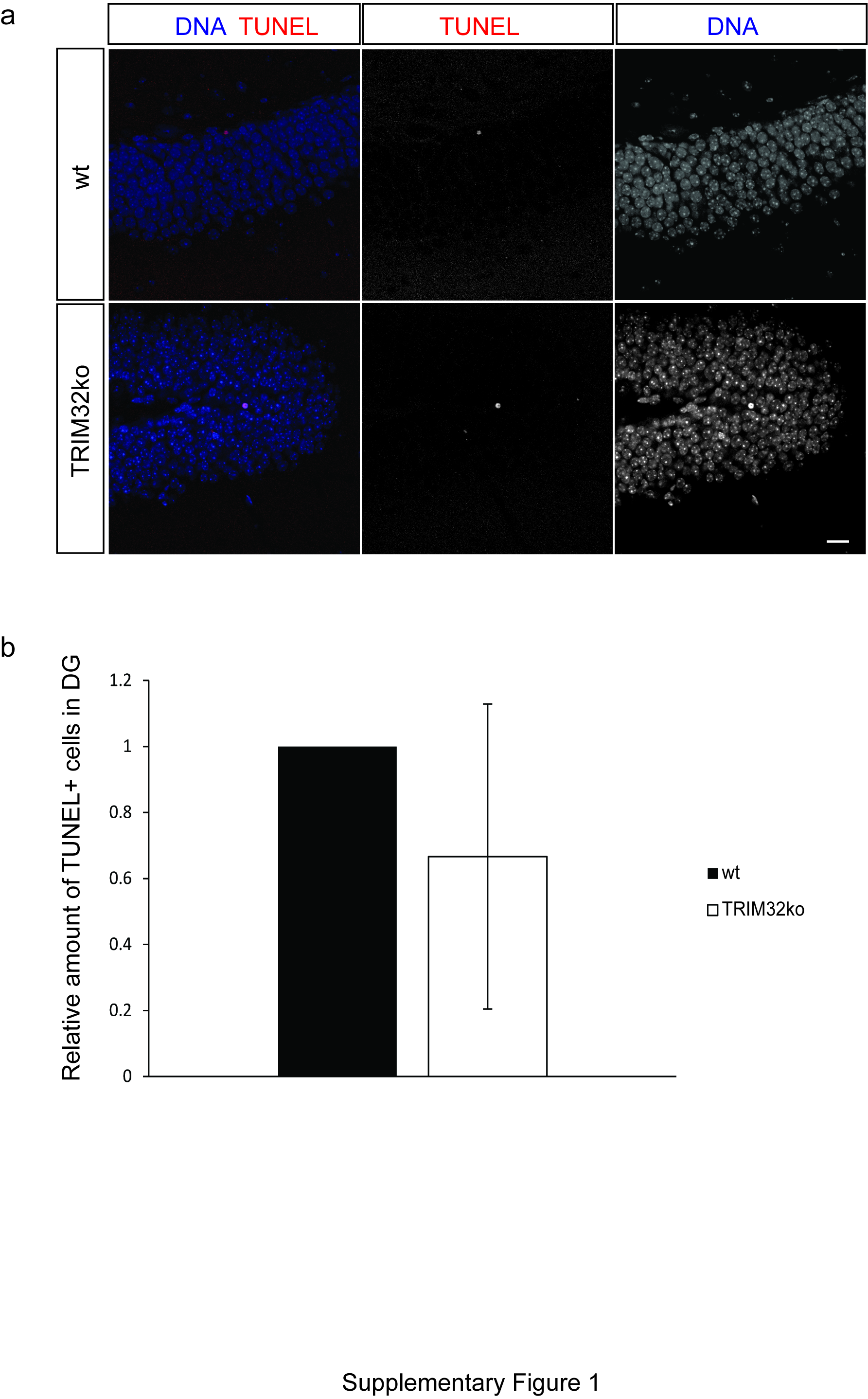

Supplement: Supplementary Figure 1 — (A) Free floating sections from wt and TRIM32 ko mouse brain stained with TUNEL and Hoechst. Shown is the dentate gyrus of the hippocampus. Scale bar = 20 μm. (B) Quantification of TUNEL+ cells in dentate gyrus of wt and TRIM32 ko mouse brain. N = 4 wt and 4 TRIM32 ko mice, n = 23 cells. [file Image1.TIF]

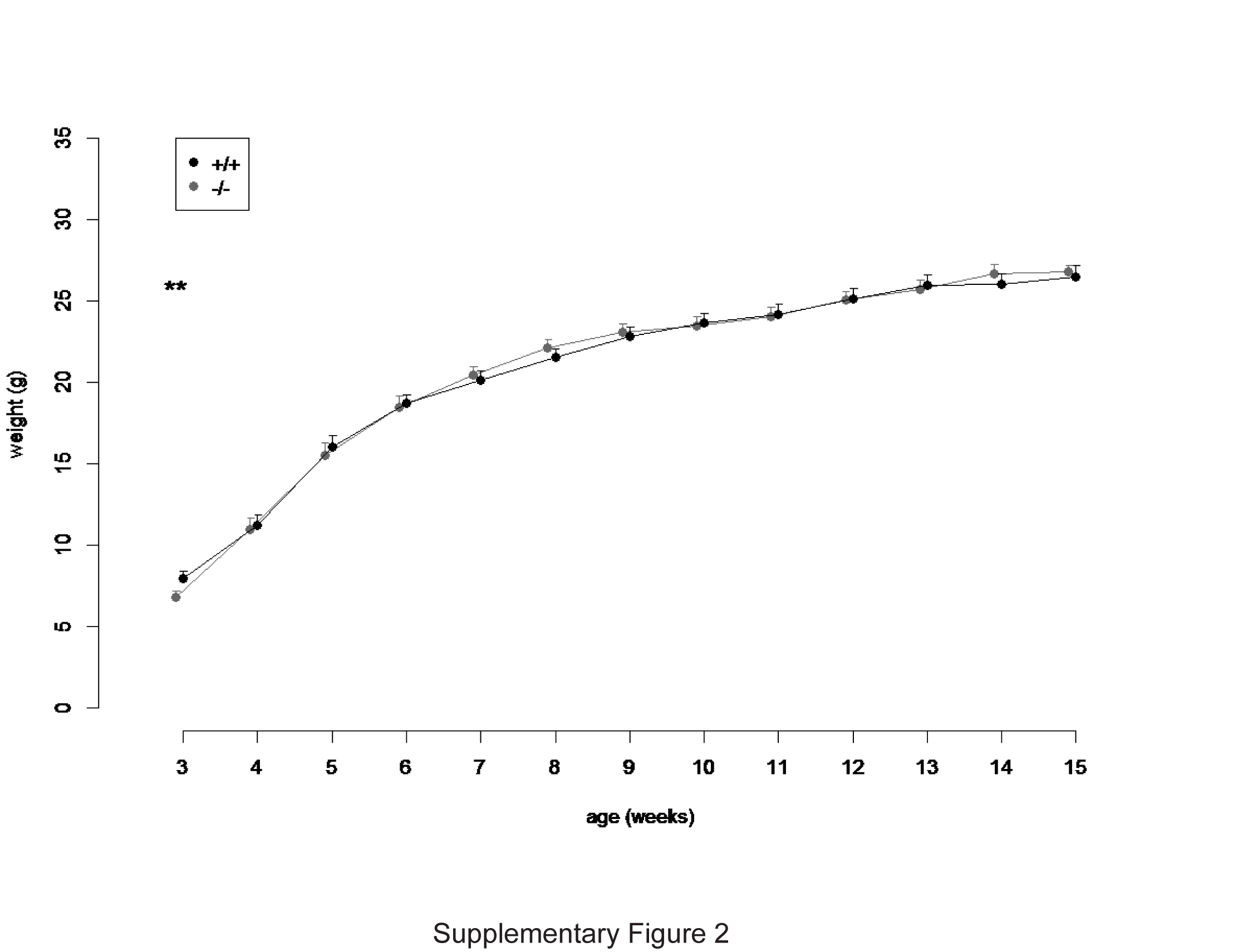

Supplement: Supplementary Figure 2 — Weight development over the course of 15 weeks. Two Sample t-test, t = 2.743, df = 53, p-value = 0.0083. [file Image2.TIF]

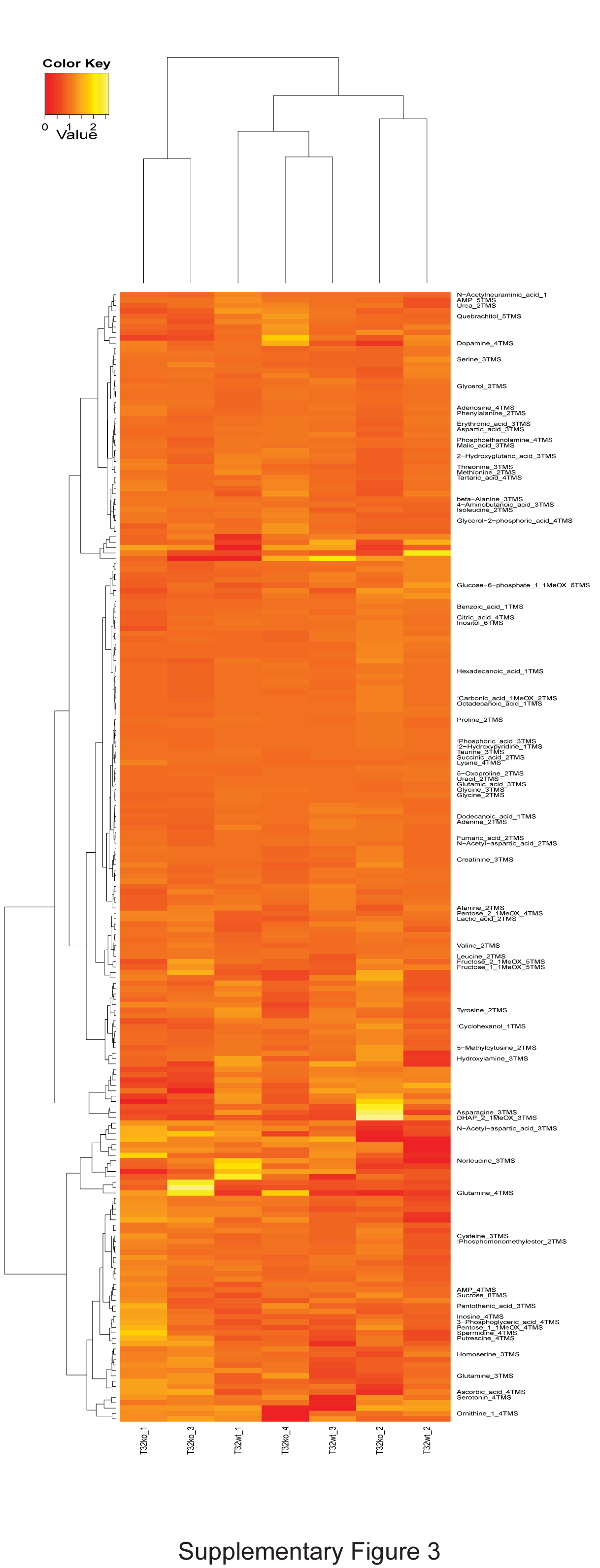

Supplement: Supplementary Figure 3 — Heatmap showing relative levels of metabolites in wt and TRIM32 ko mice. Data were calculated as medians. Colors represent relative changes of concentration in wt and TRIM32 ko brain tissue. Unlabeled rows represent unknown metabolites. Metabolome clusters consist of samples with similar concentration profiles. Three wt and 4 ko animals were used for analysis. [file Image3.TIF]

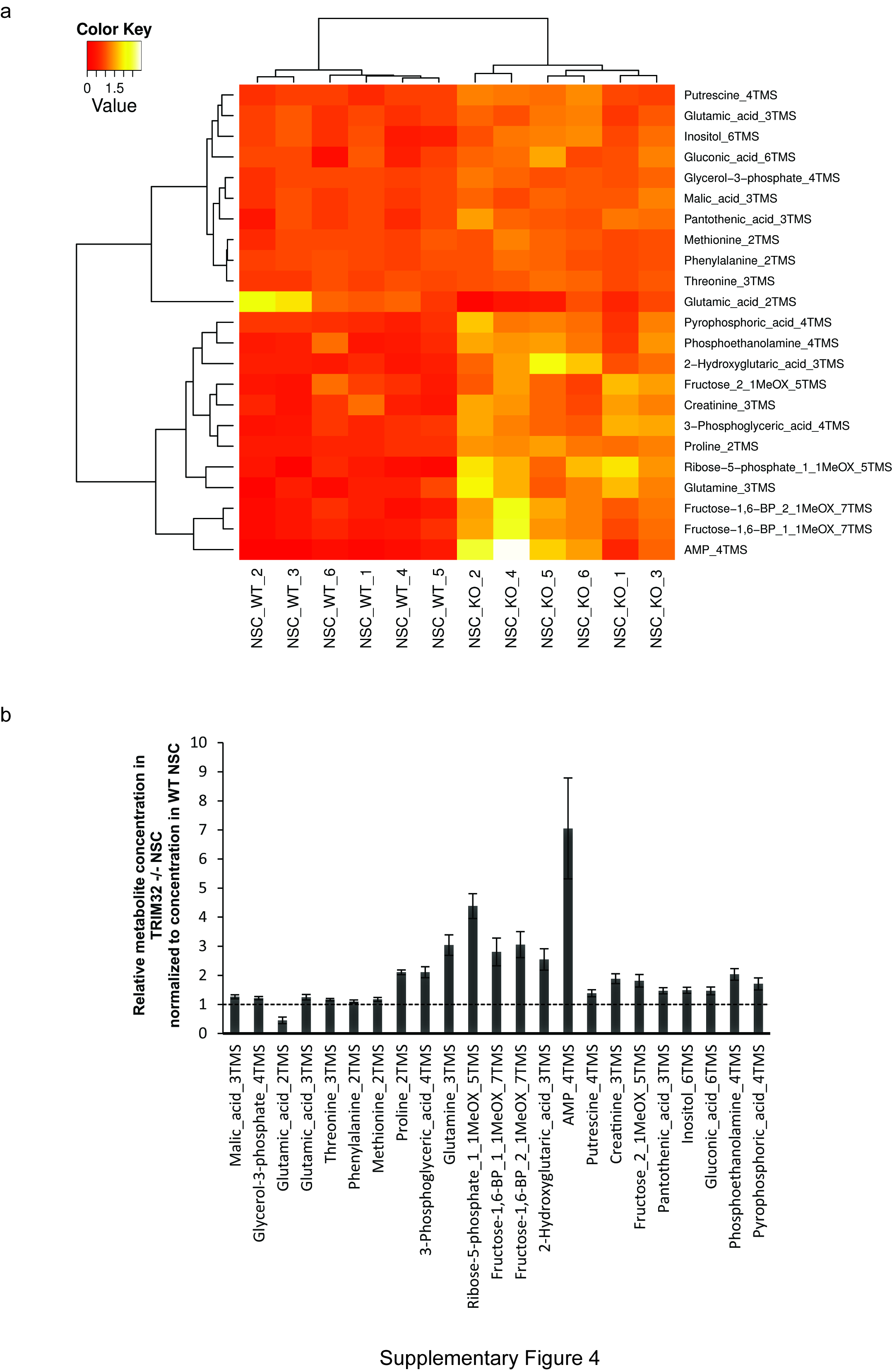

Supplement: Supplementary Figure 4 — (A) Hierarchical cluster analysis of significantly changed metabolite levels in NSCs (wild type vs. TRIM32 knock-out). The statistical significance and robustness of differences in metabolite levels (Welch's t-test, p < 0.05, n = 6) was evaluated by leave-one-out cross-validation. (B) Bar diagram showing significantly changed metabolite levels in NSCs (wild type vs. TRIM32 ko). Statistical analysis see (A), data were calculated as means with standard error of the mean and values of TRIM32 ko mice were normalized to wt values. [file Image4.TIF]
